# Clinical Course and Prognosis of Isolated Terminal Ileal Ulcers

**DOI:** 10.34172/aim.2023.66

**Published:** 2023-08-01

**Authors:** Xinjue He, Yue Chen, Xinxin Zhou, Chao Lu

**Affiliations:** ^1^Department of Gastroenterology, The First Affiliated Hospital, College of Medicine, Zhejiang University, Hangzhou, China; ^2^Department of Cardiology, The First Affiliated Hospital, College of Medicine, Zhejiang University, Hangzhou, China

**Keywords:** Endoscopy, Ileal diseases, Prognosis

## Abstract

**Background:**

Isolated terminal ileal ulcers (ITIUs) may be a clue to hidden intestinal diseases. However, there are no specific guidelines for ITIUs.

**Methods:**

We retrospectively screened subjects undergoing colonoscopy at The First Affiliated Hospital, Zhejiang University from March 2014 to July 2019, and included patients with ITIUs in the study. Some patients underwent further examination of the entire small intestine. Subsequently, patients with undetermined ITIUs received empiric therapy or observational follow-up. At least one year after baseline colonoscopy, telephone follow-up was performed to collect prognostic information.

**Results:**

A total of 120 (0.3%) patients undergoing colonoscopy in our center were found with ITIUs. Fourteen patients underwent further examination of the entire small intestine, six of whom were found with additional small bowel ulcers, but it did not significantly help the diagnosis. We obtained follow-up information from 41 undiagnosed patients. Over an average follow-up of 35.4 months, there was no significant difference in the prognosis of patients receiving empiric therapy or observational follow-up. The clinical and endoscopic outcomes improved or remain unchanged in most patients. In logistic regression analysis, gender, age, chief complaint, number of ulcers, and follow-up strategy had no significant impact on prognosis.

**Conclusion:**

Patients with nonspecific ITIUs usually improve without any therapy, and observational follow-up may be a reasonable strategy.

## Introduction

 The terminal ileum is 30–40 cm of the ileum at the distal end of the small intestine and terminates at the ileocecal sphincter. It is a common site for small bowel diseases, such as Crohn’s disease (CD),^1^ intestinal tuberculosis^2,3^ and lymphoma,^4^ as well as adenocarcinoma, drug-induced enteropathy and Behcet’s disease,^5^ which can manifest as small ulcers in the terminal ileum. Although isolated terminal ileal ulcers (ITIUs) are only occasionally observed during colonoscopy, they are likely a clue to these diseases and should be given more attention.

 In colonoscopy, ileal intubation is a marker of complete examination, especially when cecal intubation is uncertain.^6-8^ Although terminal ileal intubation can be difficult, some techniques are available when standard maneuvers fail.^9^ It has been shown that terminal ileal intubation and biopsy can improve the diagnosis of terminal ileum lesions, especially for patients with abdominal pain.^10^ However, there are no specific guidelines for the diagnosis and management of ITIU as it is usually occult, asymptomatic or with nonspecific symptoms, and lacks evidence for early diagnosis.

 In this study, we screened patients with ITIUs by colonoscopy and collected their histopathological findings. In addition, we followed the patients and analyzed the clinical course and prognosis of ITIUs.

## Materials and Methods

###  Study Design and Population

 We retrospectively screened subjects who underwent colonoscopy at The First Affiliated Hospital, College of Medicine, Zhejiang University from March 2014 to July 2019, and included the patients with ITIUs (only ulcers in the terminal ileum, no lesions in the colon or ileocecal valve).^5^ It was not appropriate or possible to involve patients or the public in the design, or conduct, or reporting, or dissemination plans of our research. Informed consent was obtained from all patients for inclusion in the study. All authors had access to the study data and reviewed and approved the final manuscript.

 Patients who met the following criteria were excluded: (1) history of related chronic intestinal disease at baseline, including inflammatory bowel disease (IBD), intestinal tuberculosis, lymphoma, colorectal cancer and Behcet’s disease; (2) endoscopic diagnosis (including histopathological evidence) of the above diseases; (3) history of colorectal surgery; (4) recent (within 6 months) use of non-steroidal anti-inflammatory drugs, corticosteroids or immunosuppressants; (5) oral or genital ulcers suggesting Behcet’s disease.

 Eligible patients may undergo some helpful laboratory tests for diagnosis, and some of them underwent further examination of the entire small intestine, including small bowel capsule endoscopy (SBCE), computed tomographic enterography (CTE), and double-balloon enteroscopy (DBE). Except for patients who were successfully diagnosed, the undiagnosed patients received follow-up management, that is, empiric therapy or observational follow-up. At least one year after the baseline colonoscopy, telephone follow-up was performed for patients with undiagnosed ITIUs to collect prognostic information about their clinical symptoms and colonoscopic re-examination.

###  Data Collection

 We recorded the patients’ gender, age, clinical symptoms, and the endoscopic features and histopathological results of ITIUs. For patients with follow-up information, data on further examination and follow-up management (empiric therapy or observational follow-up) were also collected, most importantly including the prognosis vis-a-vis clinical symptoms and endoscopic findings.

###  Statistical Analysis

 All data were analyzed using SPSS 23.0 (IBM Inc., Chicago, IL). Continuous variables were expressed as mean ± standard deviation, and the differences between two groups were compared using Student’s *t* test (based on normal distribution and homogeneity of variances); categorical variables were expressed as number (percentage), and the differences between two groups were compared using chi-square test or Fisher’s exact test as appropriate (if no more than 20% of cells with a theoretical frequency T < 5 and no cells with a T < 1, chi-square test was used; otherwise Fisher’s exact test was used). Logistic regression analysis was performed to assess whether potential influencing factors, including gender, age, chief complaint, number of ulcers, and follow-up strategy affect the clinical or endoscopic prognosis of ITIUs. A *P* value < 0.05 was considered statistically significant.

## Results

**Figure 1:**
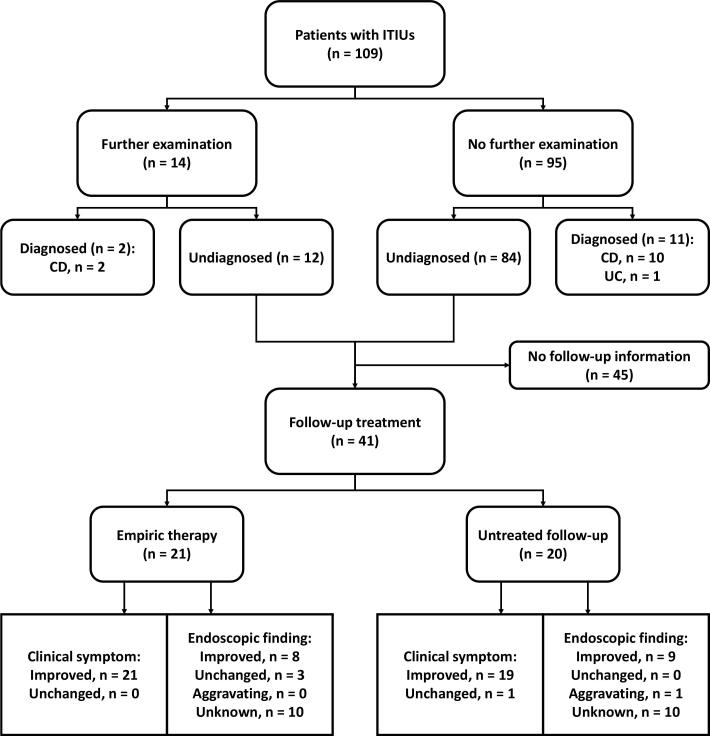
Flowchart of follow-up. CD, Crohn's disease; ITIU, isolated terminal ileal ulcer; UC, ulcerative colitis

 Among the 48 000 individuals who underwent colonoscopy in the First Affiliated Hospital, College of Medicine, Zhejiang University from March 2014 to July 2019, a total of 120 patients were found with ITIUs, with a detection rate of 0.3%. Of these patients, nine had a history of potentially related bowel disease (including five IBDs, two intestinal tuberculosis and two colorectal cancers), one was diagnosed with lymphoma based on typical histopathological evidence, and one was suspected as Behcet’s disease due to simultaneous oral ulcers. Excluding the patients with explainable ITIUs, we followed 109 eligible patients with undetermined ITIUs at baseline. The flowchart of follow-up is shown in Figure 1.

###  Baseline Characteristics of Patients

Table 1 shows the baseline characteristics of 109 eligible patients, including 73 males (67.0%), with an average age of 44.4 years. Among them, 7 patients (6.4%) referred to the hospital for health examination, 35 (32.1%) for abdominal pain, 25 (22.9%) for diarrhea, 3 (2.8%) for constipation, 10 (9.2%) for hematochezia, and 30 (27.3%) for other reasons, including abdominal discomfort or distension, dyspepsia, weight loss, anemia, fever, and other non-intestinal disorders.

**Table 1:** Baseline Characteristics of Patient with Isolated Terminal Ileal Ulcers

**Characteristics**	**ITIU Patients (n=109)**
Gender (male)	73 (67.0%)
Age	44.4 ± 15.0
Chief complaint	
Health examination	7 (6.4%)
Abdominal pain	35 (32.1%)
Diarrhea	25 (22.9%)
Constipation	3 (2.8%)
Hematochezia	10 (9.2%)
Others*	29 (26.6%)
Number of ulcers (Multiple)	40 (36.7%)
Ulcer pathology (Chronic nonspecific inflammation)	95 (87.2%)

ITIU, isolated terminal ileal ulcer. *Other complaints include abdominal discomfort or distension, dyspepsia, weight loss, anemia, fever, and other non-intestinal disorders.

 Regarding the endoscopic features of ITIUs, 69 patients (63.3%) had single ulcers and 40 (36.7%) had multiple ulcers. Except for the patients with unbiopsied ulcers (12.8%), the histopathology of ulcers in other patients (87.2%) was chronic nonspecific inflammation.

###  Further Examination and Diagnosis

 Shortly after colonoscopy, 14 patients underwent further SBCE, CTE, or DBE to examine the entire small intestine. Eight (57%) of them only had terminal ileal ulcers, while the other (43%) were found to have additional small bowel ulcers, two of whom were diagnosed with CD. In addition, 95 patients did not undergo these examinations, with or without some helpful laboratory tests, 11 (12%) of whom received a definite diagnosis, including 10 cases of CD and one case of ulcerative colitis (UC). There was no significant difference in the diagnostic rate between patients with or without further examination (14% vs. 12%, *P* = 0.673; Table 2). Finally, a total of 13 patients were diagnosed with a definite disease, including 12 CDs and one UC.

**Table 2:** Diagnostic Rate of Further Examination in Patient with Isolated Terminal Ileal Ulcers

**Diagnosis**	Further Examination* **(n=14)**	**No Further Examination** **(n=95)**	***P***
Undiagnosed	12 (86%)	84 (88%)	0.673
Diagnosed	2 (14%)	11 (12%)	
CD	2	10	
UC	0	1	

CD, Crohn's disease; UC, ulcerative colitis. *Further examinations include small bowel capsule endoscopy (SBCE), computed tomographic enterography (CTE), and double-balloon enteroscopy (DBE) to examine the entire small intestine.

###  Follow-up Management and Prognosis

 Thereafter, the remaining 96 undiagnosed patients received follow-up management, in the form of either empiric therapy (including 5-ASA, probiotics or 5-ASA plus probiotics) or observational follow-up. We obtained the follow-up information on clinical symptoms and colonoscopic re-examination in 41 patients at least one year after the baseline colonoscopy. As shown in Table 3, over an average follow-up of 35.4 months, there was no significant difference in the prognosis of patients receiving empiric therapy or observational follow-up. The clinical symptoms and endoscopic findings improved or remain unchanged in most patients of the two groups. Only one patient progressed from a single ulcer to multiple small ulcers during observational follow-up. In logistic regression analysis, gender, age, chief complaint, number of ulcers, and follow-up strategy had no significant effect on clinical or endoscopic prognosis (Table 4).

**Table 3:** Prognosis of Undiagnosed Patients Receiving Empiric Therapy or Observational Follow-up

**Prognosis**	Empiric Therapy* **(n=21)**	**Observational Follow-up** **(n=20)**	***P***
Clinical symptom			0.488
Improved	21 (100.0%)	19 (95.0%)	
Unchanged	0 (0.0%)	1 (5.0%)	
Endoscopic finding			0.258
Improved	8 (38.1%)	9 (45.0%)	
Unchanged	3 (14.3%)	0 (0.0%)	
Aggravating	0 (0.0%)	1 (5.0%)	
Unknown	10 (47.6%)	10 (50.0%)	

*Empiric therapy includes 5-ASA, probiotics, and 5-ASA plus probiotics.

**Table 4:** Logistic Regression Analysis of Potential Factors Influencing the Clinical or Endoscopic Prognosis of ITIUs

**Factor**	**Clinical Outcome**			**Endoscopic Outcome**		
	**Hazard Ratio**	**95% Ci**	***P***	**Hazard Ratio**	**95% CI**	***P***
Gender						
Female	1.000			1.000		
Male	0.776	0.000	1.000	2.013	0.061-66.034	
Age	2.968	0.000	0.998	0.972	0.862-1.097	
Chief complaint						
Health examination	1.000			1.000		
Abdominal pain	9.446	0.000	1.000	1.863	0.000	1.000
Diarrhea	565.732	0.000	1.000	0.000	0.000	0.999
Constipation	3137853.19	0.000	0.999	0.000	0.000	0.999
Hematochezia	0.000	0.000	0.999	0.000	0.000	0.999
Others*	0.000	0.000	0.999	0.566	0.000	1.000
Number of ulcers						0.670
Single	1.000			1.000		
Multiple	9.079	0.000	1.000	2.321	0.048-111.971	
Treatment						0.454
Observational follow-up	1.000			1.000		
Empiric therapy^#^	0.000	0.000	0.999	3.169	0.154-65.040	

*Other complaints include abdominal discomfort or distension, dyspepsia, weight loss, anemia, fever, and other non-intestinal disorders. ^#^Empiric therapy includes 5-ASA, probiotics, and 5-ASA plus probiotics.

## Discussion

 In our study, 0.3% of patients who underwent colonoscopy in our center were found to have ITIUs. Among them, 11.9% were diagnosed with IBDs, and there was no difference in the diagnostic rate between patients with or without further examination of the entire small intestine. Subsequently, for undiagnosed patients, there was no difference in prognosis between empiric therapy or observational follow-up. Most patients in both groups had improved clinical symptoms and endoscopic findings, or remained unchanged. In summary, ITIUs usually improve or limit themselves even without any therapy.

 As previously reported, ITIUs are occasionally observed in asymptomatic individuals, and most could resolve without empiric therapy. Even if the ulcer persists, it rarely progresses or causes any symptoms.^5^ In our study, we found that even in symptomatic individuals, ITIUs were sporadic, and most of them improved or self-limited without any therapy. Altogether, ITIUs may be a relatively benign natural history with no serious events and do not necessarily require empiric therapy or frequent colonoscopy.

 The cause of ulcers in the terminal ileum is not entirely clear. It may be related to bacterial colonization, infection and induction of immune response in the terminal ileum, leading to mucosal damage. It has been reported to be one of the earliest manifestations of certain diseases, such as CD. Patients with aphthous ulcers may develop typical CD later.^11^ However, Courville et al found that despite the chronic characteristics of ileal biopsy, isolated asymptomatic ileitis did not progress to overt CD over long-term follow-up.^12^ In our study, after baseline colonoscopy or further examinations (with or without helpful laboratory tests), patients with a successful diagnosis were most likely to be CDs, and there was no difference in the diagnostic rate between patients with and without small intestine examinations. No new CD patients were found during follow-up. These findings indicate that there are rarely missed diagnoses based on the available diagnostic methods, and small intestine examinations have a limited role in diagnosis, but may be helpful for severity assessment.

 There were some limitations in our study. First, due to the low detection rate (0.3%) of terminal ileal ulcers, only 109 eligible patients were included in this study, and the follow-up information was incomplete. Second, although the patients were diagnosed as ITIUs by colonoscopy, only a few patients underwent further examination of the entire small intestine. In this study, 6 of the 14 patients who underwent further examination were found to have additional small bowel ulcers, but its role in diagnosis seemed to be limited. Third, the study population came from a single center, and we followed up for an average of 3 years. In future studies, prospective multicenter studies with long-term follow-up are needed, as disease progression may vary between populations and take a long time.

 In summary, patients with ITIUs usually improve without any therapy, and even if the ulcer persists, it rarely progresses. These findings suggest that for patients with undetermined ITIUs based on routine diagnostic methods, we may choose observational follow-up rather than empiric therapy. However, we still recommend further diagnosis and intervention for patients at high risk of certain diseases. Of course, prospective multicenter studies with long-term follow-up are needed in the future.

## Conclusion

 Patients with ITIUs usually experience natural improvement without treatment, and they seldom deteriorate even if the ulcer persists. These findings suggest that opting for observational follow-up, rather than empirical therapy, may be a reasonable approach for patients with ITIUs when a definitive diagnosis cannot be achieved through conventional diagnostic methods.

## References

[1] Baumgart DC, Sandborn WJ (2012). Crohn’s disease. Lancet.

[2] Kedia S, Das P, Madhusudhan KS, Dattagupta S, Sharma R, Sahni P (2019). Differentiating Crohn’s disease from intestinal tuberculosis. World J Gastroenterol.

[3] Makharia GK, Srivastava S, Das P, Goswami P, Singh U, Tripathi M (2010). Clinical, endoscopic, and histological differentiations between Crohn’s disease and intestinal tuberculosis. Am J Gastroenterol.

[4] Catassi C, Bearzi I, Holmes GK (2005). Association of celiac disease and intestinal lymphomas and other cancers. Gastroenterology.

[5] Chang HS, Lee D, Kim JC, Song HK, Lee HJ, Chung EJ (2010). Isolated terminal ileal ulcerations in asymptomatic individuals: natural course and clinical significance. Gastrointest Endosc.

[6] Rees CJ, Bevan R, Zimmermann-Fraedrich K, Rutter MD, Rex D, Dekker E (2016). Expert opinions and scientific evidence for colonoscopy key performance indicators. Gut.

[7] Rex DK, Schoenfeld PS, Cohen J, Pike IM, Adler DG, Fennerty MB (2015). Quality indicators for colonoscopy. Am J Gastroenterol.

[8] Powell N, Knight H, Dunn J, Saxena V, Mawdsley J, Murray C (2011). Images of the terminal ileum are more convincing than cecal images for verifying the extent of colonoscopy. Endoscopy.

[9] Sakata S, Stevenson AR, Naidu S, Hewett DG (2017). Techniques for terminal ileal intubation at colonoscopy when standard maneuvers fail. Am J Gastroenterol.

[10] Jeong SH, Lee KJ, Kim YB, Kwon HC, Sin SJ, Chung JY (2008). Diagnostic value of terminal ileum intubation during colonoscopy. J Gastroenterol Hepatol.

[11] O’Brien CL, Kiely CJ, Pavli P (2018). The microbiome of Crohn’s disease aphthous ulcers. Gut Pathog.

[12] Courville EL, Siegel CA, Vay T, Wilcox AR, Suriawinata AA, Srivastava A (2009). Isolated asymptomatic ileitis does not progress to overt Crohn disease on long-term follow-up despite features of chronicity in ileal biopsies. Am J Surg Pathol.

